# Simultaneously enhanced dielectric properties and through-plane thermal conductivity of epoxy composites with alumina and boron nitride nanosheets

**DOI:** 10.1038/s41598-021-81925-x

**Published:** 2021-01-28

**Authors:** Zhengdong Wang, Guodong Meng, Liangliang Wang, Liliang Tian, Siyu Chen, Guanglei Wu, Bo Kong, Yonghong Cheng

**Affiliations:** 1 School of Mechanical and Electrical Engineering, Xi’an University of Architecture and Technology, Xi’an, 710055 China; 2 State Key Laboratory of Electrical Insulation and Power Equipment, Center of Nanomaterials for Renewable Energy, Xi’an Jiaotong University, Xi’an, 710049 China; 3 Shaanxi Key Laboratory of Nano Materials and Technology, Xi’an, 710055 China; 4 State Key Laboratory of Bio-Fibers and Eco-Textiles, Institute of Materials for Energy and Environment, College of Materials Science and Engineering, Qingdao University, Qingdao, 266071 China

**Keywords:** Chemical engineering, Electrical and electronic engineering

## Abstract

Dielectric materials with good thermal transport performance and desirable dielectric properties have significant potential to address the critical challenges of heat dissipation for microelectronic devices and power equipment under high electric field. This work reported the role of synergistic effect and interface on through-plane thermal conductivity and dielectric properties by intercalating the hybrid fillers of the alumina and boron nitride nanosheets (BNNs) into epoxy resin. For instance, epoxy composite with hybrid fillers at a relatively low loading shows an increase of around 3 times in through-plane thermal conductivity and maintains a close dielectric breakdown strength compared to pure epoxy. Meanwhile, the epoxy composite shows extremely low dielectric loss of 0.0024 at room temperature and 0.022 at 100 ℃ and 10^−1^ Hz. And covalent bonding and hydrogen-bond interaction models were presented for analyzing the thermal conductivity and dielectric properties.

## Introduction

Dielectric polymer composites with high dielectric constant and high dielectric breakdown strength are needed for many power electronic devices and power equipment under high electric field, such as energy storage and conversion devices^1,2^. These energy storage and conversion devices working at high electric field, probably suffer from not only high electric field intensity but also a great number of heat fluxes^3,4^. In this regard, dielectric polymer composites with high thermal conductivity and high glass transition temperature (Tg) and outstanding thermal stability are necessary to maintain the good performance and reliability for energy conversion devices^5–8^. Thermosetting epoxy resins have been widely used as insulating materials in electrical engineering because of their excellent thermal stability, dielectric properties and low cost, which show significant potential in the application of energy storage and conversion at high temperature. For instance, Chen et al.^9^ presented a kind of epoxy resin films by grafting asymmetric alicyclic amine-polyether amine molecular chain structure, which enhanced dielectric breakdown strength, thermal stability and charge–discharge efficiency of epoxy film capacitors for their application in electric power systems and electrical automobile industry. However, this kind of epoxy resin still has low thermal conductivity due to its amorphous structure^10^, possibly leading to the problem of heat dissipation and the partially interior high temperature in energy conversion devices. Although liquid crystal epoxy resins are obtaining more attention due to their relatively high thermal conductivity^11,12^, there are some limitations such as lower thermostability, therefore the nanocomposites are necessary to obtain high thermal conductivity.

Carbon materials have high thermal conductivity, but their are also electrically conductive^13^. BNNs has a large band gap (5.7–6 eV), high thermal conductivity in (002) or (004) crystal plane^14,15^, which is a good candidate for obtaining a material with high in-plane thermal conductivity and desirable dielectric properties^16–18^. However, through-plane thermal conductivity of BNNs is relatively low (around 30 W/m K). And the BNNs is easier to form horizontal distribution and the horizontal orientation of (002) or (004) in polymer matrix due to its two-dimensional structure and large aspect ratio^19^, benefit for the enhancement of in-plane thermal conductivity. Therefore, the enhancement of through-plane thermal conductivity is more difficult because of the much lower intrinsic through-plane thermal conductivity of BNNs and the interface thermal resistance between BNNs and epoxy matrix. In fact, some strategies have been employed to enhance thermal conductivity and maintain dielectric breakdown strength as high as possible, such as the alignment of fillers along through-plane direction^20^, design of multilayered structures^21^, and core–shell structures^22,23^, and so on. Alumina also has outstanding dielectric properties and an acceptable thermal conductivity of around 30 W/m K^24,25^. Alumina spheres and BNNs are expected to have a synergistic effect on increasing through-plane thermal conductivity by forming partial networks in through-plane direction.

Meanwhile, alumina and BNNs are also widely used as inorganic fillers to enhance the dielectric properties of polymers because of their desirable dielectric breakdown properties at high electric filed strength^26–30^. For example, Li et al. presented a kind of BNNs/c-BCB nanocomposites by using BNNs as fillers, obtaining significantly increasing dielectric breakdown strength and energy storage density at high temperature^26^. Compared to c-BCB, epoxy resins have lower cost and higher dielectric breakdown strength^9^. In particular, its low cost is more competitive for the practical application in energy storage and conversion devices. Moreover, some other strategies have been developed for high energy storage performances^31–35^.

In this work, epoxy composites were prepared by using epoxy resin as polymer matrix and two dimensional BNNs and zero dimensional alumina as inorganic fillers, which are expected to improve thermal conductivity, dielectric constant and maintain the dielectric breakdown strength of composites. The zero dimensional alumina in epoxy resin matrix can offer deep trap, effectively restricting the migration of free carriers, resulting in a further increase in dielectric breakdown strength of composites. Moreover, the interface interaction between fillers and epoxy matrix is analyzed to explain the improvement of dielectric properties and thermal transport performance due to the formation of hydrogen bonds and carbon boron oxygen bonds. This study is expected to obtain a deeper understanding about the effects of the embedded fillers with different dimensions including zero dimensional fillers and two dimensional fillers into polymer matrix and interface characteristic on thermal conductivity and dielectric properties of the polymer composites with various fillers.

## Experimental section

### Materials

BNNs with lateral size of around 1 μm was provided by XFNANO Co. Ltd, Jiangsu. Alumina microspheres with a average diameter of 200 nm were provided by Aladdin Reagent Co. Ltd, Shanghai. The bisphenol-A epoxy resin (DGEBA, EPON-828) were commercially available from Hexion Specialty Chemicals Co. Ltd, Shanghai. And methyl tetrahydrophthalic anhydrite and benzyldimethylamine were employed as the hardener and catalytic agent, respectively, which were purchased from Aladdin Reagent Co. Ltd, Shanghai.

### Fabrication of epoxy composite specimens

The epoxy composites with hybrid fillers were prepared by the following experimental procedures. Alumina and BNNs of a certain proportion were mixed into hardener solution and sonicated for forming a homogeneously dispersed suspension, which had no visible agglomerations and there is no residual fillers on the edges of beaker. Subsequently, epoxy monomers were mixed into the suspension of fillers and hardener solution and effectively dispersed by ball milling in a via planetary mixer with Zirconia balls of various sizes, which their diameters are 3 mm and 5 mm, respectively. And then the catalytic agent was dispersed by an extra ball milling for a short time. After that, the obtained suspensions were carried out a degassing treatment via a vacuum equipment at around 60 °C until no bubbles removed (about half an hour). Then the final suspensions were infunded into a mould for curing process by a precuring process at 100 °C for 120 min and subsequently post curing at 150 °C for 600 min. In this study, alumina, BNNs and epoxy were abbreviated as A, B and Ep, respectively. B/Ep composites were prepared by using various filler loading from 2 to 10% (percent by volume), which were defined as A2, A4, A6, A8 and A10, respectively. For A/B/Ep, the loading alumina was same at 6%, and those of BNNs were 2 and 4%, respectively (defined as A6 + B2 and A6 + B4).

### Characterization

Thermal diffusivity (*α*) and specific heat (*C*_p_) were tested by using laser flash analyzer (LFA 467, NETZSCH). The densities (*ρ*) of specimens were obtained by measuring their volume and mass. Thermal conductivity (*TC*) values of specimens were obtained by a typical equation: *TC* = *α* × *C*_p_ × *ρ*. The specimen was sliced into quadrate with the length of around 1 cm on the side. Subsequently, a layer of graphite was sprayed onto the surface of samples and measured at different temperature. For dielectric measurements, a layer of gold were sputtered onto the surface of samples used as electrodes via an auto sputtering equipment. Dielectric constant, dielectric loss and alternating current conductivity of samples were measured by broadband dielectric analyzer (CONCEPT80, Nov Tech Co, Germany). The values of dielectric breakdown strength were tested by a classical breakdown system with two ball–ball electrodes. The slice of specimens was held by two spherical electrodes and then was immersed into electrically insulating oil. Subsequently, step-voltage measurement with a rate of 2 kV/s was carried out until breakdown. Thermal gravimetric analysis (TGA) was carried out from room temperature to 800 °C at the temperature rate of 10 °C/min in nitrogen atmosphere. Differential scanning calorimetry (DSC, 200 F3, NETZSCH, Germany) was carried out at temperature from 25 to 200 °C at a heating rate of 10 °C/min under a nitrogen atmosphere to study glass transition temperature (Tg) of samples. Infrared spectrums of the alumina and BNNs were obtained by a fourier transform infrared spectrometer (FT-IR, IN1+IZ10, Nicolet) for further analyzing the groups on the surface of fillers. Surface morphologies of fillers in the epoxy composites were observed by a SEM equipment (FEI QUANTA F250). The phase structure of materials was examined by X-ray diffraction using Bruker D2 PHASER diffractometer.

## Results and discussion

### Characterization of functionalized BNNs

#### Morphology and structure analysis of epoxy composites

**Figure 1:**
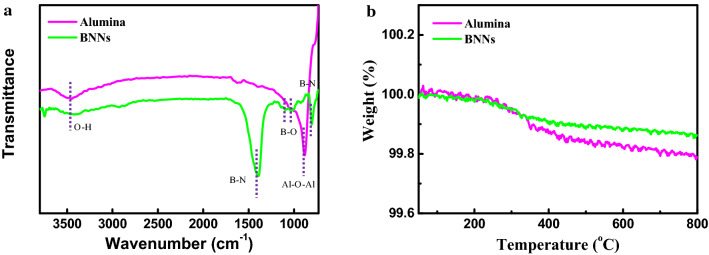
(**a**) FTIR spectrum of, and (**b**) TGA curves of the alumina spheres and the BNNs powder.

The Fourier transform infrared (FTIR) spectrum of the alumina spheres and the BNNs powder is shown in Fig. 1a. The typical B–N stretching peak and bending peak of BNNs are observed at 1378 cm^−1^ and 795 cm^−1^, respectively, steaming from the in-plane sp^2^ B–N bonds and the out-of plane B–N–B bonds. Moreover, the BNNs show distinct peaks of hydroxyl group at 3446 cm^−1^ and the multiple peaks of B–O bonds at 1093 and 1021 cm^−1^ due to the formation of B–OH groups via water absorption and hydrolysis of defect sites on the edges of BNNs^36–38^. By the similar principle, aside from the absorption peak of Al–O–Al (883 cm^−1^), the peak of hydroxyl group on the surface of the alumina spheres can be observed at 3452 cm^−1^. The thermogravimetric analysis (TGA) (Fig. 3a) indicates that there is an observing weight loss in the region of 250–400 ℃, which is attributed to thermal degradation of hydroxyl group joint on the edges or surface of fillers. These results suggest the hydroxyl groups have already existed, and then form the hydrogen bonding interaction with the hydroxyl or amine groups in the polymer matrices.

**Figure 2:**
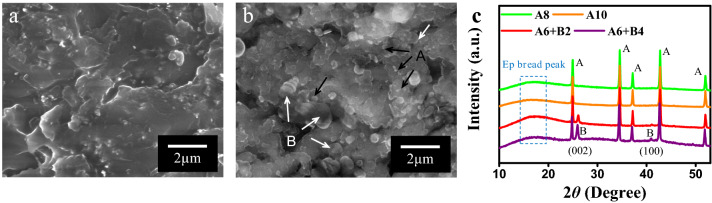
Cross-section SEM morphology of (**a**) A8, (**b**) A6 + B2, and (**c**) XRD patterns of epoxy composites with individual or hybrid fillers.

Figure 2 compares the surface morphologies and XRD patterns of the epoxy composites with different fillers, respectively. The fractured surface of epoxy composite with alumina spheres is showed in Fig. 2a. As seen from SEM image of the A/Ep composite, the alumina particles shows uniform distribution and compact interface structures, which are no obvious clusters or voids in the composite. The compact interface and homogeneous distribution could be explained by the hydrogen bonding interaction between alumina and epoxy resin matrix. Figure 2b shows the surface morphology of the A6 + B2 by SEM image, which seems more rough in comparison with those of A/Ep. The interface between fillers and epoxy matrix looks more indistinct, which is attributed to the large specific surface area and high surface energy of ultra-thin BNNs aside from the hydrogen bonding and covalent bonding interaction, leading to the more intense combination and conglutination.

Moreover, alumina microspheres will be a connecting bridge among separated BNNs, forming some partial networks to increase thermal transport performance. More importantly, the alternating distribution structure of various fillers with different dimension can show a synergistic effect on the improvement of dielectric breakdown and discharge. Figure 2c shows the XRD patterns of epoxy composites with individual or hybrid fillers. Compared to patterns of epoxy composites with individual filler, there are two obvious extra diffraction peak corresponding to (002) crystal plane and (100) crystal plane of BNNS, indicating that BNNS were successfully embedded. Moreover, The epoxy composites with various fillers show the typical bread peak of crosslinking epoxy molecular with amorphous structure due to curing reaction. More remarkably, A/Ep and A/B/Ep exhibit the different peak intensity of curing epoxy. More specifically, A6 + B2 and A6 + B4 have stronger peak intensity of curing epoxy, suggesting the role of boron free radicals from BNNs by ball milling and sonicating, which contribute to the crosslinking reaction of epoxy.

### Through-plane thermal conductivity (TC) and thermal stability analysis

**Figure 3:**
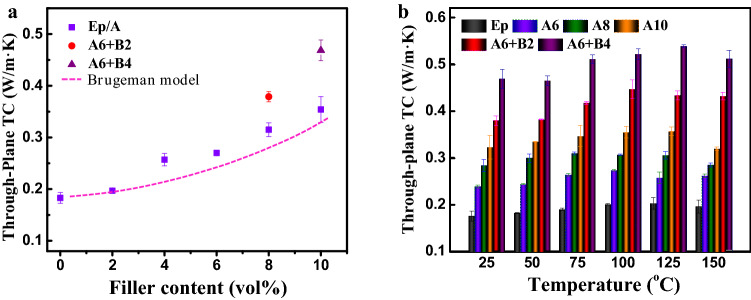
(**a**) Experimental and theoretical results of through-plane TC at room temperature of epoxy and its composites, (**b**) through-plane TC at various temperature of epoxy and its composites.

Generally speaking, all of the Morphology, structure and dispersion of filler and interface resistance have a significant effect on thermal transport performance of polymer nanocomposites^16,17,39–41^. Experimental values and theoretical evaluation of through-plane TC of the pure epoxy and its composites at room temperature were shown in Fig. 3a, through-plane TC values of the epoxy composites increase with the increment of filler content. The TC of pure epoxy was 0.18 W/m K, consistent with the previous study^42,43^. Through-plane TC of the A2 is 0.20 W/m K, rather close to that of pure epoxy. It is worth noting that the through-plane TCof A/B/Ep composites are distinctly higher than those of the A/Ep composites. For instance, through-plane TC of the A8 and A10 are 0.32 and 0.35 W/m K, respectively. And those of the A6 + B2 and A6 + B4 are 0.38 and 0.47 W/m K, respectively.

The results may be ascribed to the following reasons. (1) Intrinsic thermal conductivity of BNNs is higher than that of alumina microspheres. (2) Alumina microspheres and BNNs have a synergistic effect on the enhancement of through-plane TC. More precisely, alumina microspheres serve as connecting bridges for separated BNNs, favorable for the formation of partial thermal transport networks due to the difference of their morphologies. (3) Interface interaction between epoxy and BNNs, forming covalent bonds in addition to hydrogen bonds, is stronger than that between epoxy and alumina.

In order to further analyze thermal transport performance of the epoxy composites, Bruggeman model was used to predict their thermal conductivity values^44^. Bruggeman model was shown as follows:

1 $${1 - }\varphi_{{\text{f}}} = \frac{{K_{{\text{f}}} - K_{{\text{c}}} }}{{K_{{\text{f}}} - K_{{\text{m}}} }}\left( {\frac{{K_{{\text{m}}} }}{{K_{{\text{c}}} }}} \right)^{{\frac{{1}}{{3}}}}$$

where *K*_c_, *K*_m_, and *K*_f_ are the thermal conductivity of the epoxy composites, epoxy matrix, and alumina filler, respectively, and *Φ*_f_ are the volume fraction of the filler. As seen from Fig. 3a, the predicted values of thermal conductivity are slightly lower to the experimental results of A/Ep composites. This is mainly because themal conductivity is predicted based on the isolated fillers in this model. This means that the interaction between fillers was taken into consideration, not to mention the hydrogen bonds in the interface. A/B/Ep composites have higher thermal conductivity due to the synergistic effect and strong covalent linkages (C–O–B bonds) in the interface besides hydrogen bonds.

Figure 3b shows the thermal conductivity of epoxy composites at various temperatures. It can be seen that the thermal conductivity of epoxy composites show slightly increase with increasing temperature until 125 ℃. And thermal conductivity shows slight decrease at 150 ℃, which mainly is attributed to the relaxation of epoxy molecular, when the testing temperature is more than glass transition temperature (T_g_) of epoxy composites, as shown in the Fig. S1 and Table S1. It means that the crosslinked epoxy networks were destroyed at high temperature, suppressing phonon conduction and heat transport.

### Dielectric properties

**Figure 4:**
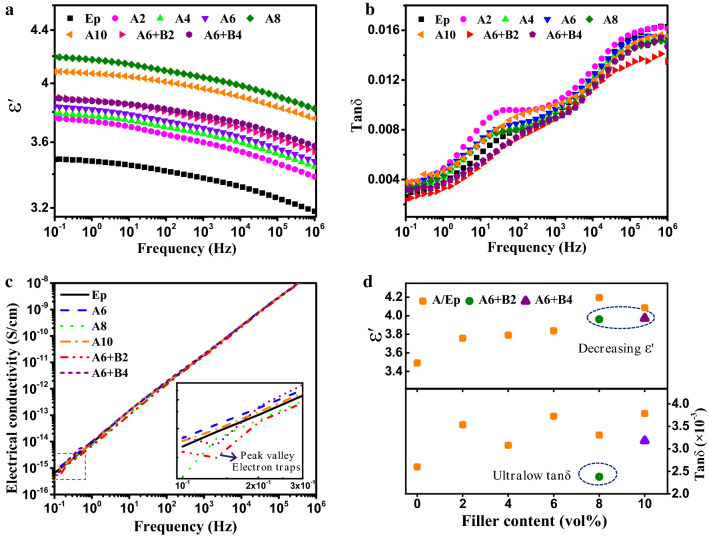
The dependence of (**a**) dielectric constant, (**b**) dielectric loss, (**c**) electrical conductivity of pure epoxy and its composites on frequency. (**d**) Dielectric constant and loss plots of pure epoxy and its composites.

As expected, the dielectric constant increased with increasing filler content in epoxy resin matrix from 10^–1^ to 10^6^ Hz at room temperature (25 ℃). The dielectric constant and dielectric loss of epoxy composites with different fillers and loading were shown in Fig. 4a,b. Dielectric polarization and loss of epoxy composites are mainly attributed to 3 parts as follows: (1) the electronic displacement polarization and conduction loss caused by embedding inorganic fillers and impurity ions; (2) the relaxation polarization and loss from some polar groups in epoxy matrix; (3) interface polarization and loss between inorganic fillers and epoxy matrix. Based on Fig. 4a, the dielectric constant of all epoxy composites slightly decrease with increasing frequency. It can be mainly ascribed to the lower influence of relaxation polarization at the high frequency due to the mismatch between relaxation polarization and change frequency of electrical filed. Therefore, the dielectric constant of epoxy composites decreased as frequency increased. The dielectric constant of pure epoxy resin was around 3.5 at 50 Hz, and the dielectric constant of A/Ep composites were higher compared to that of pure epoxy. This is attributed to the higher dielectric constant of alumina and interface polarization, which two key factors for the increment of dielectric constant of epoxy composites. Compared to those of A/Ep composites, the dielectric constant of A/B/Ep composite systems is obviously lower due to the lower intrinsic dielectric constant and good electrically insulating property of BNNs. More importantly, interface between BNNs and epoxy forms chemical interface bonding (covalent bonds), which is much stronger interface layer than hydrogen bonding interaction between alumina and epoxy. The strong and tight interface constraints the movement of epoxy molecualr and relaxation polarization at the interface, resulting in lower dielectric constant. Moreover, this phenomenon can also be explained by the dipole orientation polarization from Debye relaxation theory. The dielectric properties of polymer with semiconducting or insulating fillers have a close correlation with the electrical properties of inorganic fillers^45,46^, effecting on the migration and accumulation of free carriers at the interface between fillers and polymer matrix. BNNs have a large band gap and high electrical resistivity, suppressing migration of carriers and charges, leading to the lower electronic displacement polarization.

The dependence of dielectric loss of epoxy composites on frequency was shown in Fig. 4b. It can be seen that there is a peak of dielectric loss for A/Ep composites at the frequency range from 10 to 100 Hz. In other words, the dielectric loss increases rapidly twice with increasing frequency from 0.1 to 50 Hz and 10^3^ to 10^6^ Hz. In contrast, A/B/Ep composites show a relatively slow increase in dielectric loss with the increment of frequency. Meanwhile, the dielectric loss of A/B/Ep composites are also distinctly lower than those of A/Ep composites. For instance, the dielectric loss of A6 + B2 is only 0.0024 at 0.1 Hz, which is lower than that of neat epoxy, as shown in Fig. 4d. Even at power frequency of 50 Hz, its dielectric loss is also very low (0.0068). It can be ascribed to lower interface loss and conduction loss of A/B/Ep composites. Interface in the A/B/Ep composites include chemical interface and overlapping interface. The chemical interface is formed from hydrogen-bond and covalent bond interaction between BNNs and epoxy matrix. Moreover, the overlapping interface can be obtained by interface interaction of adjacent fillers, reducing the free volume of epoxy molecular. Both chemical interface and overlapping interface will contribute to forming better and tighter interface, suppressing interface loss and conduction loss. Figure 4c shows the dependence of electric conductivity on frequency at room temperature of epoxy composite systems. The electric conductivity increases as tested frequency increases. The dielectric constant of the epoxy composites with BNNs is lower than that of pure epoxy from 10^–1^ to 10^6^ Hz. It can be attributed to the high intrinsic resistance of BNNs and the interface layer between epoxy matrix and fillers based on the mutil-core model^47^, restraining the direct migration of carriers and prolonging the transfer router of carriers in composites. Meanwhile, the intercalation of BNNs introduces great number of electron traps, resulting in the formation of peak valley of electrical condution. Therefore, it declares that the intercalating BNNs play key role in blocking the conduction of carries in the epoxy composites.

Dielectric breakdown strength of materials is an important parameter to evaluate the insulation characteristic of dielectric materials. The dielectric breakdown strength values are analyzed by two-parameter Weibull distribution given by Eqs. (2) and (3):

2 $$P\left( E \right) = 1 - {\text{exp}}\left[ { - \left( {\frac{E}{{E_{0} }}} \right)^{\beta } } \right],$$

where *P*(*E*) is the cumulative probability of electrical failure, *E* is the experimental breakdown strength, *β* is the shape parameter which evaluates the scattering of data, and *E*_0_ represents the characteristic dielectric breakdown strength at the cumulative failure probability of 63.2%. *P* is calculated as follows:

3 $$P_{{\text{i}}} = \frac{i - 0.44}{{n + 0.25}},$$

where *i* is the *i*-th result when *E* values are sorted in ascending order of breakdown strength data, and *n* is the number of total data points.

**Figure 5:**
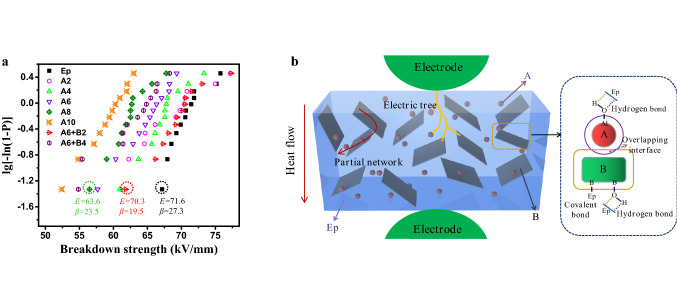
(**a**) Dielectric breakdown strength of pure epoxy and its composites based on Weibull distribution. (**b**) Schematic diagram of breakdown processing in epoxy composites with hybrid fillers.

Figure 5a shows dielectric breakdown strength of pure epoxy and its composites based on weibull distribution. It can be seen from the Fig. 5a that intercalation of the sub-micron alumina spheres into epoxy matrix leads to a slight decrease in the dielectric breakdown strength. However, intercalation of BNNs into A/Ep composites distinctly enhances the dielectric breakdown strength values. For example, the dielectric breakdown strength of the A6 + B4 is 64.5 kV/mm, which increases by 6.8% compared to that of the A10. Moreover, the A6 + B2 show dielectric breakdown strength values of over 70 kV/mm, which is in close proximity to that of pure epoxy. It can be mainly attributed to 3 reasons as follows. First, interface of A/Ep composites will be relatively loose due to the sub-micron size of alumina spheres. And this loose interface will form more free volume of epoxy molecular and come about free charge under high electric field leading to a decreasing dielectric breakdown strength. In contrast, interface in A/B/Ep composites will be tighter due to large aspect ratio of BNNs, which reduces the free volume of epoxy and suppresses migration of free charge. Second, BNNs with layered structure will prolong the dielectric breakdown paths, resulting in more winding paths or termination for the germination and development of electric trees, as illustrated in Fig. 5b. Thirdly, in general, breakdown damage of dielectrics always was induced by thermal accumulation breakdown because electric breakdown happened within extremely short time (nanosecond time scale). As illustrated in Fig. 5b, intercalation of BNNs into A/Ep composite systems can form partial thermal conductive network, increasing effectively thermal transport capacity and suppressing probability of thermal accumulation breakdown. Moreover, the interface characteristic between polymer and fillers is considered as another important factor for explaining the enhancement of dielectric performance. As mentioned above, The A/B/Ep composites have hydrogen-bond and covalent bond interaction in the interface, contribute to a stronger interface. Meanwhile, the adjacent fillers will form overlapping interface, restraining mobility of epoxy molecular and suppressing conduction and dielectric loss. Finally, the dielectric breakdown strength is enhanced.

**Figure 6:**
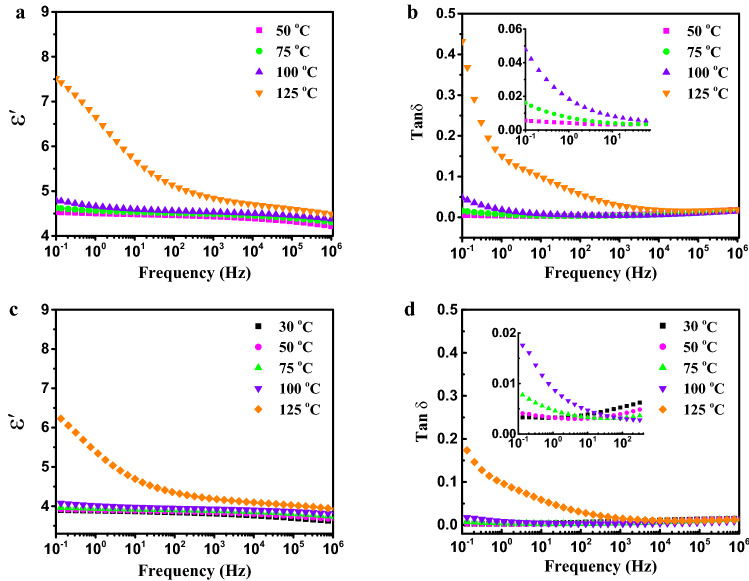
The dependence of dielectric properties for (**a**,**b**) pure epoxy, and (**c**,**d**) A6 + B2 at different temperature on frequency.

To further study the dielectic properties and explore their potential for application at high temperatures, the temperature dependence of dielectric properties of pure epoxy and A/B/Ep composites were measured. As shown in Fig. 6a,b, pure epoxy shows a weak temperature dependence of the dielectric constant and dielectric loss under 75 ℃. The dielectric constant and loss of pure epoxy begin to increase quickly at low frequency (< 10^2^ Hz) when the tested temperature is at 100 ℃ or more. In particular, the dielectric constant and loss of pure epoxy at 125 ℃ and 10^–1^ Hz are 7.7 and 0.45, respectively. Compared to the pure epoxy, A6 + B2 shows no obvious increase in dielectric constant and loss at 100 ℃ and still remains relatively low dielectric constant (6) and dielectric loss (0.16) at 125 ℃ and 10^–1^ Hz, as shown in Fig. 6c,d, mainly attributed to the relatively low relaxation polarization of A6 + B2 at high temperature. The alumina and BNNs in A6 + B2 will form good interface by chemical bonding and overlapping interface, restricting the movement of the polymer molecular chain and increasing heat dissipation, leading to the higher glass transition temperature (See Table S1). Therefore, A6 + B2 shows lower relaxation polarization and loss. Dielectric loss of A6 + B2 still remains a low range (0.022) at 100 ℃ and 10^–1^ Hz, which is desirable for energy storage and energy conversion in capacitors and the practical application at high temperature.

### Dielectric properties and thermal conductivity discussion based on interface interaction

**Figure 7:**
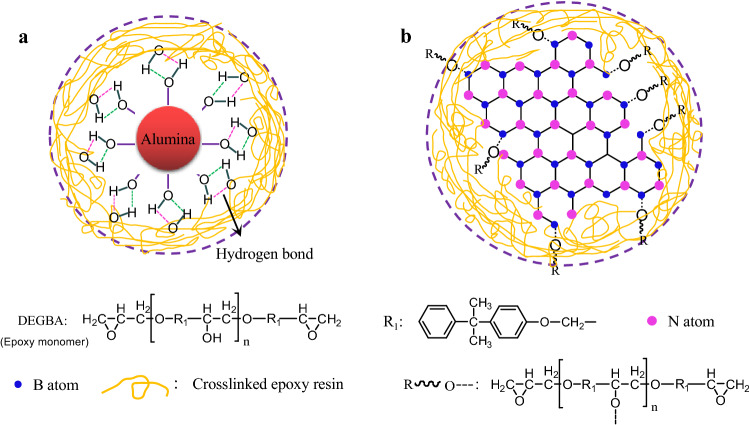
Interfacial structures of fillers with epoxy molecular (**a**) alumina, and (**b**) BNNs.

In addition to the synergistic effect of alumina and BNNs, the other important mechanism for enhancing dielectric breakdown strength and high-temperature dielectric properties is the interface interaction between fillers and polymer matrix. In the work, the surface of alumina microspheres have a large number of hydroxyl groups (see Fig. 1a,b), which could form hydrogen bonding with the hydroxyl or amine groups in crosslinked epoxy resin, as illustrated in Fig. 7a. The hydrogen bonding interaction is considered as a crucial reason to improve the compatibility between fillers and epoxy matrix, and thus could ameliorate the deterioration of dielectric properties caused by the intercalation of fillers. According to the previous reference^48–51^, the edges of BNNs have a large number of unstable and broken B–N bonds. These broken chemical bonds especially for B atoms are identified as B free radicals^50,51^, which are rather active to combine the hydroxyl or amine groups. Therefore, th edges of BNNs exist some hydroxyl groups by absorbing water molecular. More notably, the broken chemical bonds or B free radicals could be obtained during the process of epoxy composites by sonicating and ball milling. These new B free radicals is feasible to combine the hydroxyl or amine groups in epoxy resin matrix and hardener during pre-curing and post-curing process under high temperature, as illustrated in Fig. 7b. And then the formation of stable C–O–B bonds could enhance the interface interaction and compatibility between BNNs and epoxy, resulting in the excellent breakdown strength, dielectric properties at high temperature and good thermal stability as the Fig. S2 and Table S1 show.

**Figure 8:**
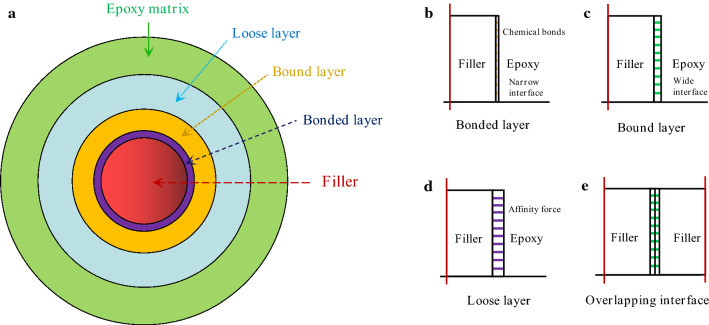
Interface strucrures in a similar multi-core model for epoxy composites.

Since nanodielectric material was proposed by Lewis^52^, the interface issues between nanofillers and polymer matrix have already obtained lots of attention and extensive study^22,29,53^. And some theoretical and empirical models about the interface were presented^47,54^. In particular, multi-core model presented by T Tanaka was generally accepted by research scholars^47^. In this work, a similar multi-core model is constructed by utilizing the hydrogen bond and C–O–B chemical bond, as illustrated in Figs. 7 and 8. In addition to the central filler, there are three layers including bonded layer, bound layer and loose layer from inside-out in the model, respectively. Compared to bound layer, the interface zone in bonded layer is narrow, as illustrated in Fig. 8b,c, which is mainly derived from one or two of covalent bonds, ionic bonds, hydrogen bonds, and van der Waals force. It is worth noting that the bonding strength in the bonded layer has a significant effect on the formation and structure of the bound layer and loose layer. This means that the strong chemical bonds such as covalent or ionic bonding in the bonded layer can form a tight and good interface in the bound layer and loose layer. To be more specific, when there are no hydrogen bond or C–O–B covalent bond between fillers (alumina or BNNs) and epoxy matrix, the interface zone of loose layer is bigger in the case of the only affinity, and the interphase in loose layer is considered to be separated, as shown in Fig. 8d. In contrast, if there existed hydrogen bond or C–O–B covalent bond, these bonds will constraint the mobility of the relaxed epoxy molecular and reduce the free volume in the loose layer. Indeed, in addition to the chemical interface from hydrogen bonds or, ionic or covalent bonds, the strong interface strength also can be obtained by forming a overlapping interface zone due to the synergistic effect of fillers. The free volume in loose layer will be obviously reduced in the overlapping interface zone of bound or bonded layers, as illustrated in Fig. 8e. For example, as the bound or bonded layers of alumina overlap with those of BNNs, resulting in the reduction of loose layer and improvement of dielectric breakdown strength. The enhanced interface strength obtained by chemical bonds and synergistic effect of fillers also have a positive influence on the increment of thermal conductivity of polymer composites by motivating phonon conduction and reducing thermal resistance based on some previous studies^55–60^. In this regard, the study on the interface characteristic of nanocomposite dielectric materials is very promising for addressing the challenges in interface thermal resistance and improving dielectric properties.

The energy density of capacitors with linear dielectrics can be expressed by the following equation *U*_ed_ = 0.5 ε_0_ *ε*_r_ *E*^2^. Therefore, the energy density (*U*_ed_) of materials mainly depends on the dielectric constant (*ε*_r_) and dielectric breakdown strength (*E*). Compared to pure epoxy, the *E* of A6 + B2 is rather close and its *ε*_r_ is relatively higher. The energy density of A6 + B2 shows an increasing by 11.3% compared to that of pure epoxy, and other epoxy composites show slight decrease in energy storage capability due to the decrease of dielectric breakdown strength. Even though, these epoxy composites still shows strong application potential due to the distinct increase of through-plane thermal conductivity, which can be capable of working at the high temperature. Moreover, it is observed that the both pure epoxy and its composites show extremely low dielectric loss characteristic, which is very benefit for the charge–discharge efficiency and good stability^61^. It is clear that epoxy and its composites by intercalating BNNs and alumina are promising for the energy storage and energy conversion devices at relatively high temperature because of its extremely low dielectric loss and ultra-high dielectric breakdown strength. It’s worth noting that energy density of the epoxy and its composites with hybrid fillers could be enhanced significantly by preparing the much thinner epoxy films^9^ due to the significant increase in dielectric breakdown strength for thin-film materials, and its composites films with high energy density and thermal conductivity are expected in our future work.

## Conclusions

In summary, we prepared two epoxy-based composites using alumina or the hybrid fillers of alumina and BNNs as fillers. The thermal conductivity and dielectric properties of epoxy composites were studied. Compared to pure epoxy and A/Ep composites, the A/B/Ep show good synergistic effect on the enhancement of thermal transport property and maintaining high dielectric breakdown strength and low dielectric loss. In particular, the A6 + B2 exhibits relatively higher thermal conductivity and energy density. For instance, through-plane thermal conductivity of A6 + B2 increase of around 3 times compared to those of pure epoxy. Meanwhile, the A6 + B2 shows ultra-low dielectric loss (0.022) at high temperature (100 ℃) and 10^–1^ Hz compared to that (0.05) of pure epoxy under same testing condition. Moreover, the glass transition temperature and thermal decomposition temperature of A6 + B2 also is much higher than that of pure epoxy and A/Ep composites, explaining that the A/B/Ep composites have lower dielectric loss at high temperature. Therefore, the simple strategies of epoxy with hybrid fillers show good synergistic effect, interface interaction and outstanding comprehensive properties, which is desirable for applications in microelectronic devices and power equipments under high electric field such as energy storage and conversion devices, insulated gate bipolar translator (IGBT) and gas insulated substation (GIS) etc.

## Supplementary Information

Supplementary Information.
