# Supplementary material for: Simultaneously enhanced dielectric properties and through-plane thermal conductivity of epoxy composites with alumina and boron nitride nanosheets
Source: Sci Rep. 2021 Jan 28;11:2495. doi: 10.1038/s41598-021-81925-x (PMC7844292; doi:10.1038/s41598-021-81925-x)
Supplement: Supplementary file 1 — Supplementary Information. [file 41598_2021_81925_MOESM1_ESM.doc]

**Simultaneously Enhanced Dielectric properties and through-plane thermal conductivity of epoxy composites with alumina and boron nitride nanosheets**

Zhengdong Wang^a,b,c^*, Guodong Meng^b^*, Liangliang Wang^a,c^, Liliang Tian^b^, Siyu Chen^b^, Guanglei Wu^d^, Bo Kong^b^, Yonghong Cheng^b^*

^a^ School of Mechanical and Electrical Engineering, Xi'an University of Architecture and Technology, Xi'an, 710055, China;

^b^ Center of Nanomaterials for Renewable Energy, State Key Laboratory of Electrical Insulation and Power Equipment, Xi'an Jiaotong University, Xi'an, 710049, China;

^c^ Shaanxi Key Laboratory of Nano Materials and Technology, Xi'an, 710055, China

^d^ Institute of Materials for Energy and Environment, State Key Laboratory of Bio-fibers and Eco-textiles, College of Materials Science and Engineering, Qingdao University, Qingdao 266071, P. R. China;

* Correspondence: wangzhengdong@xauat.edu.cn (Z. Wang); gdmengxjtu@xjtu.edu.cn (G. Meng)；cyh@xjtu.edu.cn (Y. Cheng)

**Figure S1** DSC curves of pure epoxy and its composites

**Figure S2.** TGA curves of pure epoxy and its composites

Thermal stability of the epoxy composites was studied by TGA, as shown in Figure 3d. No distinct mass loss is observed as the temperature is less than 250 °C because of the excellent thermal stability of epoxy. Epoxy composites start to decompose as testing temperature is over 250 °C. It’s worth noting that the A/Ep composites exhibit an increase in initial decomposition temperature (IDT) in comparison with pure epoxy. And the A/B/Ep composites show higher IDT. In addition to resistance to high temperature of fillers, the main reason is that A/B/Ep composites have a tight interface between fillers and epoxy matrix by chemical bonds and overlapping interface, constrainting the mobility of epoxy molecular and increasing heat dissipation, resulting in the better thermostability.

**Table S1.** Thermal properties of pure epoxy and its composites with individual or hybrid fillers via DSC and TGA results.

| Samples | Weight loss temperature (^o^C) | Glass transition temperature (^o^C) | |
| --- | --- | --- | --- |
|  | Initial decomposition temperature | Tg (DSC) | ΔTg (DSC) |
| Pure epoxy | 322 | 128.4 | - |
| A8 | 327 | 130.5 | 2.1 |
| A10 | 323 | 131.4 | 3.0 |
| A6+B2 | 335 | 134.6 | 6.2 |
| A6+B4 | 343 | 132.2 | 3.8 |
